# Role of Social Media Marketing Activities in Influencing Customer Intentions: A Perspective of a New Emerging Era

**DOI:** 10.3389/fpsyg.2021.808525

**Published:** 2022-01-17

**Authors:** Khalid Jamil, Liu Dunnan, Rana Faizan Gul, Muhammad Usman Shehzad, Syed Hussain Mustafa Gillani, Fazal Hussain Awan

**Affiliations:** ^1^School of Economics and Management, North China Electric Power University, Beijing, China; ^2^Department of Management Sciences and Engineering, Zhengzhou University, Zhengzhou, China; ^3^Faisalabad Business School, National Textile University, Faisalabad, Pakistan

**Keywords:** social media marketing activities, social identification, satisfaction, continuance intention, participate intention, purchase intention

## Abstract

The aim of this study is to explore social media marketing activities (SMMAs) and their impact on consumer intentions (continuance, participate, and purchase). This study also analyzes the mediating roles of social identification and satisfaction. The participants in this study were experienced users of two social media platforms Facebook and Instagram in Pakistan. A self-administered questionnaire was used to collect data from respondents. We used an online community to invite Facebook and Instagram users to complete the questionnaire in the designated online questionnaire system. Data were collected from 353 respondents, and structural equation modeling (SEM) was used to analyze the data. Results show that SMMAs have a significant impact on the intentions of users. Furthermore, social identification mediates the relationship between social media activities and satisfaction, and satisfaction mediates the relationship between social media activities and the intentions of users. This will help marketers how to attract customers to develop their intentions. This is the first novel study that used SMMAs to address the user intentions with the role of social identification and satisfaction in the context of Pakistan.

## Introduction

There has been tremendous growth in the use of social media platforms such as WhatsApp, Instagram, and Facebook over the past decade (Chen and Qasim, 2021). People are using these platforms to communicate with one another, and popular brands use them to market their products. Social activities have been brought from the real world to the virtual world courtesy of social networking sites. Messages are sent in real time which now enable people to interact and share information. As a result, companies consider social media platforms as vital tools for succeeding in the online marketplace (Ebrahim, 2020). The use of social media to commercially promote processes or events to attract potential consumers online is referred to as social media marketing (SMM). With the immense rise in community websites, a lot of organizations have started to find the best ways to utilize these sites in creating strong relationships and communications with users to enable friendly and close relationships to create online brand communities (Ibrahim and Aljarah, 2018).

Social media marketing efficiently fosters communications between customers and marketers, besides enabling activities that enhance brand awareness (Hafez, 2021). For that reason, SMM remains to be considered as a new marketing strategy, but how it impacts intentions is limited. But, to date, a lot of research on SMM is focused on consumer’s behavior, creative strategies, content analysis and the benefits of user-generated content, and their relevance to creating virtual brand communities (Ibrahim, 2021).

New channels of communication have been created, and there have been tremendous changes in how people interact because of the internet developing various applications and tools over time (Tarsakoo and Charoensukmongkol, 2020). Companies now appreciate that sharing brand information and consumer’s experience is a new avenue for brand marketing due to the widespread use of smartphones and the internet, with most people now relying on social media brands. Therefore, developing online communities has become very efficient. Social groups create a sense of continuity for their members without meeting physically (Yadav and Rahman, 2017). A community that acquires products from a certain brand is referred to as a virtual brand community. Customers are not just interested in buying goods and services but also in creating worthwhile experiences and strong relationships with other customers and professionals. So, when customers are part of online communities, there is a cohesion that grows among the customers, which impacts the market. Therefore, it is up to the companies to identify methods or factors that will encourage customers to take part in these communities (Ismail et al., 2018).

The online community’s nature is like that of actual communities when it comes to creating shared experiences, enabling social support, and attending to the members’ need to identify themselves, regardless of the similarities and variances existing between real-world communities and online communities (Seo and Park, 2018). Regarding manifestations and technology, online communities are distinct from real-life communities since the former primarily use computers to facilitate their operation. A certain brand product or service is used to set up a brand community. Brand communities refer to certain communities founded based on interactions that are not limited by geographical restrictions between brand consumers (Chen and Lin, 2019). Since consumers’ social relationships create brand communities, these communities have customs, traditions, rituals, and community awareness. The group members learn from each other and share knowledge about a product, hence appreciating each other’s actions and ideas. So, once a consumer joins a particular brand community, automatically, the brand becomes a conduit and common language linking the community members together because of sharing brand experiences (Arora and Sanni, 2019).

Based on the perspective of brand owners, most research has focused on how social communities can benefit brands. However, there are also some discussions regarding the benefits that come from brand community members according to the members themselves to analyze how social community impacts its members (Shareef et al., 2019). Consumer’s behavior is influenced by value so, when a consumer is constantly receiving value, it leads to consumer’s loyalty toward that brand. According to Alalwan et al. (2017), a valuable service provider will create loyalty to a company and enhance brand awareness. Consumer value is essentially used in evaluating social networking sites. With better and easier options to create websites coming around, most consumers are attracted to a social community to know about a company and its goods. Furthermore, operators can learn consumer’s behavior through maintaining social interactions with customers. However, the social community should have great value. It should be beneficial to the potential customers by providing them with information relevant to the brand in question. Furthermore, customers should be able to interact with one another, thus creating a sense of belonging. From that, it is evident that a brand social community’s satisfaction affects community retention and selection.

## Literature Review

### Social Media Marketing Activities

Most businesses use online marketing strategies such as blogger endorsements, advertising on social media sites, and managing content generated by users to build brand awareness among consumers (Wang and Kim, 2017). Social media is made up of internet-associated applications anchored on technological and ideological Web 2.0 principles, which enables the production and sharing of the content generated by users. Due to its interactive characteristics that enable knowledge sharing, collaborative, and participatory activities available to a larger community than in media formats such as radio, TV, and print, social media is considered the most vital communication channel for spreading brand information. Social media comprises blogs, internet forums, consumer’s review sites, social networking websites (Twitter, Blogger, LinkedIn, and Facebook), and Wikis (Arrigo, 2018).

Social media facilitates content sharing, collaborations, and interactions. These social media platforms and applications exist in various forms such as social bookmarking, rating, video, pictures, podcasts, wikis, microblogging, social blogs, and weblogs. Social networkers, governmental organizations, and business firms are using social media to communicate, with its use increasing tremendously (Cheung et al., 2021). Governmental organizations and business firms use social media for marketing and advertising. Integrated marketing activities can be performed with less cost and effort due to the seamless interactions and communication among consumer partners, events, media, digital services, and retailers *via* social media (Tafesse and Wien, 2018).

According to Liu et al. (2021), marketing campaigns for luxury brands consist of main factors such as customization, reputation, trendiness, interaction, and entertainment which significantly impact customers’ purchase intentions and brand equity. Activities that involve community marketing accrue from interactions between events and the mental states of individuals, whereas products are external factors for users (Parsons and Lepkowska-White, 2018). But even though regardless of people experience similar service activities, there is a likelihood of having different ideas and feelings about an event; hence, outcomes for users and consumers are distinct. In future marketing, competition will focus more on brand marketing activities; hence, the marketing activities ought to offer sensory stimulation and themes that give customers a great experience. Now brands must provide quality features but also focus on enabling an impressive customer’s experience (Beig and Khan, 2018).

### Social Identification

A lot of studies about brand communities involve social identification, appreciating the fact that a member of a grand community is part and parcel of that community. Social identity demystifies how a person enhances self-affirmation and self-esteem using comparison, identity, and categorization (Chen and Lin, 2019). There is no clear definition of the brand community or the brand owner, strengthening interactions between the community and its members or creating a rapport between the brand and community members. As a result, members of a community are separated into groups based on their educational attainment, occupation, and living environment. Members of social networks categorize each other into various groups or similar groups according to their classification in social networks (Salem and Salem, 2021).

Brand identification and identification of brand communities emanate from a similar process. Users can interact freely, hence creating similar ideologies about the community, alongside strengthening bonds among members, hence enabling them to identify with that community. The brand community identity can also be considered as a convergence of values between the principles of the social community and the values of the users (Wibowo et al., 2021).

According to Lee et al. (2021), members of a brand social community share their ideas by taking part in community activities to help create solutions. When customers join a brand community, they happily take part in activities or discussions and are ready to help each other. So, it is evident that social community participation is impacting community identity positively. Community involvement entails a person sharing professional understanding or knowledge with other members to enhance personal growth and create a sense of belonging (Gupta and Syed, 2021). According to Haobin Ye et al. (2021), it is high time community identity be incorporated in virtual communities since it is a crucial factor that affects the operations of virtual communities. Also, community identity assists in facilitating positive interactions among members of the community, encouraging them to actively take part in community activities (Assimakopoulos et al., 2017). This literature review suggests that social communities need members to work together. Individuals who can identify organizational visions and goals become dedicated to that virtual company.

### Satisfaction

Customer’s satisfaction involves comparing expected and after-service satisfaction with the standards emanating from accumulated previous experiences. According to implementation confirmation theory, satisfaction is a consumer’s expected satisfaction with how the services have lived up to those expectations. Customers usually determine the level of satisfaction by comparing the satisfaction previously experienced and the current one (Pang, 2021).

According to recent studies, community satisfaction impacts consumer’s loyalty and community participation. A study community’s level of satisfaction is determined by how its members rate it (Jarman et al., 2021). Based on previous interactions, the community may be evaluated. When the members are satisfied with their communities, it is manifested through joyful emotions, which affect the behavior of community members. In short, satisfaction creates active participation and community loyalty (Shujaat et al., 2021).

### Types of Intentions

A lot of studies about information and marketing systems have used continuance intention in measuring if a customer continues to use a certain product or service. The willingness of customers to continue using a good or service determines if service providers will be successful or not. According to Zollo et al. (2020), an efficient information marketing system should persuade users to use it, besides retaining previous users to guarantee continued use.

Operators of social networks must identify the reason propelling continued use of social network sites, alongside attracting more users. Nevertheless, previous studies on information systems in the last two decades have mainly concentrated on behavior–cognition approaches, for instance, the technology acceptance model (TAM), theory of planned behavior (TPB), and theory of reasoned action (TRA) with their variants (Tarsakoo and Charoensukmongkol, 2020; Jamil et al., 2021b). According to Ismail et al. (2018), perceived use and satisfaction positively impact a user’s continuance intention. The continued community members’ participation has two intentions. Continuance intention is the first one. It defines the community member’s intent to keep on using the community (Beig and Khan, 2018; Dunnan et al., 2020). Then, recommendation intention, also known as mouth marketing, describes every informal communication that takes place among community members regarding the virtual brand community. Previous studies about members of a virtual community mostly entailed the continuous utilization of information systems (Seo and Park, 2018; Sarfraz et al., 2021). Unlike previous studies, this study focuses on factors that support the continued participation of community members. So, besides determining how usage purpose affects continuance intention, the study also investigated the factors that influence users’ willingness to take part in community activities (Gul et al., 2021).

Nevertheless, it is hard to determine and monitor whether a certain action occurred (recommendation or purchase) during empirical investigations. Consumers will seek relevant information associated with their external environment and experiences when purchasing goods (Shareef et al., 2019). Once they have collected significant information, they will evaluate it, and draw comparisons from which customer’s behavior is determined. Since purchase intention refers to a customer’s affinity toward a particular product, it is a metric of a customer’s behavioral intention. According to Liu et al. (2021), the probability of a customer buying a particular product is known as an intention to buy. So, when the probability is high, it simply means that the willingness to purchase is high. Past studies consider purchase intention as a factor that can predict consumer’s behavior alongside the subjective possibility of consumer’s purchases. According to Chen and Qasim (2021), from a marketing viewpoint, if a company wants to retain its community besides achieving community targets while establishing successful marketing *via* the community, at least three objectives are needed. They include membership continuance intention, which entails members living up to their promises in the community and also the willingness to belong to the community (Yadav and Rahman, 2018; Naseem et al., 2020). On the other side, community recommendation intention entails the willingness of members to recommend or refer community members to other people who are not members (Jamil et al., 2021a; Mohsin et al., 2021). The next consideration is the community participation intention of a member, which involves their willingness to participate in the activities of the brand community. Unlike past literature about using information systems, this study demystified how SMMAs influence purchase intention and participation intention (Alalwan et al., 2017).

### Development of Hypotheses

People with similar interests can get a virtual platform to discuss and share ideas courtesy of social media. Sustained communication of social media allows users to create a community. Long-lasting sharing of growth and information fosters the development of strong social relationships. The information posted on social media platforms by an individual positively correlates with the followers the user has. Regarding the discussion above, we proposed the following hypothesis:

H1: Social media marketing activities (SMMAs) have a significant impact on social identification.

The study of Farivar and Richardson (2021) on users’ continuance intention confirmed that it is influenced by satisfaction after service. Social media studies are also of the thought that satisfaction significantly affects continuance intention. So, a consumer will measure the satisfaction of service after using it. Mahendra (2021) claims that satisfaction influences repurchase behavior. Repurchase intention emanates from a customer’s satisfaction with a good or service. People who have similar interests may interact and cooperate in a virtual world *via* social media platforms. A community on social media may be formed by regularly connecting with people and exchanging information with them. Members benefit from long-term information and growth exchanges that enable them to create strong social relationships. A lot of studies have pointed out that repurchase intention and customer’s satisfaction are positively and highly related. Besides, marketing studies noted that satisfactory experience after using a product would impact the intention of future repurchase. Hence, we proposed the following hypothesis:

H2: SMMAs have a significant impact on satisfaction.

The study by Suman et al. (2021) on American consumer’s behavior suggested that members taking part in community activities (meetups, discussion, and browsing) influence their brand-associated behavior. According to Di Minin et al. (2021), the brand identity of a consumer has a positive impact on satisfaction. Consumers capitalize on online communities to share their experiences and thoughts about a grand regularly and easily (Sirola et al., 2021). These experiences make up the customer to brand experiences and establish a sense of belonging, trust, and group identity. In a nutshell, this study suggests that identity will enable members to recognize their community, hence confirming that members have similar experiences and feelings with a particular brand and feel united in the group (Shujaat et al., 2021). Strong group identity means that members are integrated closely into the brand communities and highly regard the community. Hence, we proposed the following hypothesis:

H3: Social identification has a significant impact on satisfaction.

Brand communities are beneficial in the sense that they enable sharing of marketing information, managing a community, and exploring demands (Dutot, 2020). These activities are likely to enhance consumer’s rights and increase customer’s satisfaction (Sahibzada et al., 2020). A customer who makes an online transaction will be highly satisfied with a website that provides a great experience (Koçak et al., 2021). Enhancing customer’s satisfaction, encouraging customer intentions, creating community loyalty, and fostering communication and interactions between community users are crucial to lasting community platform management (Pang, 2021). Hence, we proposed the following hypotheses:

H4: Satisfaction has a significant impact on continuance intention.
H5: Satisfaction has a significant impact on participate intention.
H6: Satisfaction has a significant impact on purchase intention.

Thaler (1985) proposed transaction utility theory, in which consumers’ willingness to spend money is influenced by their perceptions of value. Researchers such as Dodds (1991) claimed that buyers only become ready to purchase after they have established a sense of value for a product. According to Petrick et al. (2001), a product’s quality is dependent on the customer’s satisfaction. Several studies have shown that enjoyment, perceived value, and behavioral intention are all linked together. Hence, we proposed the following hypothesis:

H7: Social identification mediates the relationship between SMMA and satisfaction.

When it comes to information systems, Bhattacherjee et al. (2008) discovered that people’s continual intention is derived from their satisfaction with the system after they have used it. Studies on employee’s satisfaction in the workplace have shown that it has a substantial influence on CI. The amount of satisfaction that users have with the system that they have previously used is the most important factor in determining their CI, according to research on information system utilization intention.

In other words, the customer’s contentment with the product leads to the establishment of a desire to buy the thing again, as mentioned by Assimakopoulos et al. (2017). Numerous studies show a strong link between customer’s satisfaction and their propensity to return for another transaction. According to a lot of marketing studies, customers who have a pleasant experience with a product are more likely to repurchase it. Hence, we proposed the following hypotheses:

H8: Satisfaction mediates the relationship between social identification and continuance intention.
H9: Satisfaction mediates the relationship between social identification and participate intention.
H10: Satisfaction mediates the relationship between social identification and purchase intention.

**FIGURE 1:**
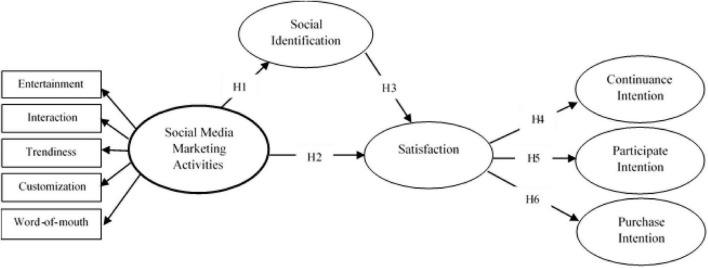
Conceptual framework.

Figure 1 shows the research framework of this study.

## Conceptual Framework

### Research Methodology

This study designed a questionnaire according to the hypotheses stated above. The participants in this study were experienced users of two social media platforms Facebook and Instagram in Pakistan. A self-administered questionnaire was used to collect data from respondents. A pilot study with 40 participants was carried out. Since providing recommendations, revisions were made to the final questionnaire to make it more understandable for the study’s respondents. To ensure the content validity of the measures, three academic experts of marketing analyzed and make improvements in the items of constructs. The experts searched for spelling errors and grammatical errors and ensured that the items were correct. The experts have proposed minor text revisions to social identification and satisfaction items and advised that the original number of items is to be maintained. This study used an online community to invite Facebook and Instagram users to complete the questionnaire in the designated online questionnaire system. Online questionnaires have the following advantages (Tan and Teo, 2000): (1) sampling is not restricted to a single geological location, (2) lower cost, and (3) faster questionnaire responses. A total of 353 questionnaires were returned from respondents. There were 353 appropriate replies considered for the final analysis.

### Measures

The study used items established from prior research to confirm the reliability and validity of the measures. All items are evaluated through 5-point Likert-type scales where “1” (strongly disagree), “3” (neutral), and “5” (strongly agree).

#### Dependent Variable

To get a response about three dimensions of intention (continuance, participate, and purchase), we used eight items adopted from prior studies;

1. Continuance intention is measured by three items from the study of Bhattacherjee et al. (2008), and the sample item is, “I intend to continue buying social media rather than discontinue its use.”
2. Participate intention is evaluated by three items from the work of Debatin et al. (2009), and the sample item is, “my intentions are to continue participating in the social media activities.”
3. Purchase intention was determined by two items adapted from the work of Pavlou et al. (2007), and the sample item is, “I intend to buy using social media in the near future.”

#### Independent Variable

To analyze the five dimensions of SMMAs, we used eleven items adopted from a prior study of Kim and Ko (2012).

1. Entertainment is determined by two items and the sample item is, “using social media for shopping is fun.”
2. Interaction is evaluated by three items, and the sample item is, “conversation or opinion exchange with others is possible through brand pages on social media.”
3. Trendiness is measured by two items, and the sample item is, “contents shown in social media is the newest information.”
4. Customization is measured by two items, and the sample item is, “brand’s pages on social media offers customized information search.”
5. Word of mouth is measured by two items, and the sample item is, “I would like to pass along information on the brand, product, or services from social media to my friends.”

#### Mediating Variables

We used two mediating variables in this study,

1. Social identification was measured with five items adopted from the prior study of Bhattacharya and Sen (2003), and the sample item is, “I see myself as a part of the social media community.”
2. Satisfaction was evaluated with six items adopted from the study of Chen et al. (2015), and the sample item is, “overall, I am happy to purchase my desired product from social media.”

## Results

This research employs a partial least square (PLS) modeling technique, instead of other covariance-based approaches such as LISREL and AMOS. The reason behind why we pick PLS-SEM is that it is most suitable for confirmatory and also exploratory research (Hair Joe et al., 2016). Structural equation modeling (SEM) has two approaches, namely covariance-based and PLS-SEM (Hair et al., 2014). PLS is primarily used to validate hypotheses, whereas SEM is most advantageous in hypothesis expansion (Podsakoff et al., 2012). A PLS-SEM-based methodology would be done in two phases, first weighing and then measurement (Sarstedt et al., 2014). PLS-SEM is ideal for a multiple-order, multivariable model. To do small data analysis is equally useful in PLS-SEM (Hair et al., 2014). PLS-SEM allows it easy to calculate all parameter calculations (Hair Joe et al., 2016). The present analysis was conducted using SmartPLS 3.9.

### Model Measurement

Table 1 shows this study model based on 31 items of the seven variables. The reliability of this study model is measured with Cronbach’s alpha (Hair Joe et al., 2016). As shown in Table 1, all items’ reliability is robust, Cronbach’s alpha (α) is greater than 0.7. Moreover, composite reliability (CR) fluctuates from.80 to.854, which surpassed the prescribed limit of 0.70, affirming that all loadings used for this research have shown up to satisfactory indicator reliability. Ultimately, all item’s loadings are over the 0.6 cutoff, which meets the threshold (Henseler et al., 2015).

**TABLE 1:** Inner model evaluation.

Variables		Item loading	AVE	CR	α
Continuance intention	CI1	0.887	0.794	0.880	0.712
	CI2	0.756			
	CI3	0.881			
Customization	Cust1	0.759	0.652	0.851	0.741
	Cust2	0.878			
	Cust3	0.679			
	Cust4	0.844			
Entertainment	E1	0.781	0.687	0.865	0.762
	E2	0.884			
	E3	0.719			
	E4	0.861			
Interaction	Int1	0.840	0.753	0.859	0.671
	Int2	0.762			
	Int3	0.756			
	Int4	0.724			
	Int5	0.858			
	Int6	0.799			
Participant intention	PI1	0.872	0.894	0.934	0.825
	PI2	0.940			
	PI3	0.913			
Purchase intention	PuI1	0.896	0.652	0.850	0.739
	PuI2	0.822			
Social identification	SI1	0.775	0.907	0.929	0.685
	SI2	0.815			
	SI3	0.772			
	SI4	0.828			
	SI5	0.819			
Satisfaction	Satis1	0.861	0.862	0.900	0.643
	Satis2	0.778			
	Satis3	0.807			
	Satis4	0.874			
	Satis5	0.833			
	Satis6	0.808			
Trendiness	Trn1	0.852	0.900	0.952	0.909
	Trn2	0.955			
	Trn3	0.822			
	Trn4	0.952			
Word of mouth	WOM1	0.767	0.685	0.864	0.760
	WOM2	0.868			
	WOM3	0.788			
	WOM4	0.876			

**FIGURE 2:**
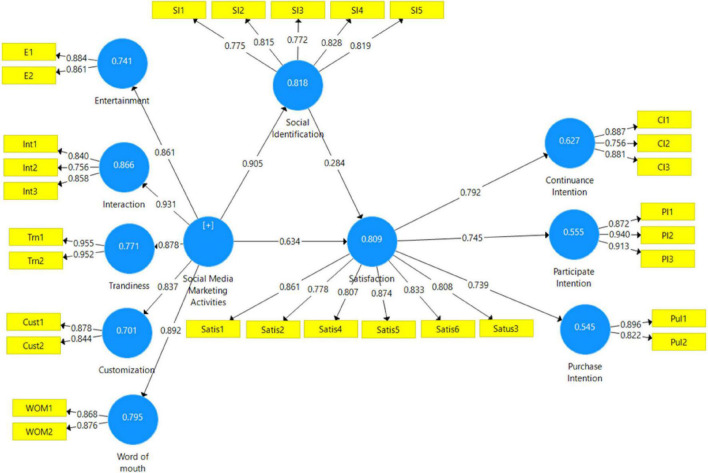
Measurement model.

The Cronbach’s alpha value for all constructs must be greater than 0.70 is acceptable (Hair et al., 2014). All the values of α are greater than 0.7 as shown in Table 1 and Figure 2.

Convergent validity is measured by CR and AVE, and scale reliability for each item (Hair Joe et al., 2016). The scholar says that CR and AVE should be greater than 0.7 and 0.5, respectively. By utilizing CR and average variance extracted scores, convergent validity was estimated (Fornell and Larcker, 1981). As elaborated in Table 3, the average variance extracted scores of all the indicators are greater than 0.50 and CR is higher than.70 which is elaborating an acceptable threshold of convergent validity and internal consistency. It is stated that a value of CR, that is, not less than 0.70, is acceptable and evaluated as a good indicator of internal consistency (Sarstedt et al., 2014). Moreover, average variance extracted scores of more than 0.50 demonstrate an acceptable convergent validity, as this implies that a specific construct with greater than 50% variations is clarified by the required indicators.

**TABLE 2:** A mediation analysis.

Hypotheses	Direct effect	Indirect effect	Total Effect	VIF	Decision
SMM- > SI- > SAT	B = 0.213, *t*-value = 3.570, *p* = 0.032	B = 0.257, *t*-value = 4.481, *p* = 0.000	B = 0.284, *t*-value = 5.348, *p* = 0.000	75%	Supported
SI- > SAT- > CI	B = 0.342, *t*-value = 3.435, *p* = 0.000	B = 0.225, *t*-value = 4.636, *p* = 0.000	B = 0.425, *t*-value = 6.543, *p* = 0.000	64%	Supported
SI- > SAT- > PAI	B = 0.324, *t*-value = 5.324, *p* = 0.000	B = 0.211, *t*-value = 4.338, *p* = 0.000	B = 0.439, *t*-value = 4.345, *p* = 0.000	73%	Supported
SI- > SAT- > PAI	β = 0.312, *t*-value = 3.434, *p*-value = 0.000	β = 0.213, *t*-value = 5.437, *p*-value = 0.000	B = 0.431, *t*-value = 5.932, *p* = 0.000	73%	Supported
					

**TABLE 3:** Discriminant validity.

Fornell–Larcker criterion							Heterotrait–monotrait (HTMT) ratios						
	CI	PI	PUI	Sat	SI	SMMA		CI	PI	PUI	Sat	SI	SMMA
CI	0.844						CI	0.813					
PI	0.682	0.908					PI	0.854	0.873				
PUI	0.622	0.714	0.860				PUI	0.765	0.825	0.789			
Sat	0.792	0.745	0.739	0.827			Sat	0.847	0.869	0.786	0.876		
SI	0.778	0.769	0.798	0.818	0.802		SI	0.785	0.854	0.734	0.786	0.768	
SMMA	0.823	0.879	0.839	0.821	0.605	0.770	SMMA	0.759	0.769	0.945	0.804	0.846	0.876

*CI, continuance intention; PI, participate intention; PUI, purchase intention; Sat, satisfaction; SI, social identification; SMMA, social media marketing activities.*

This study determines the discriminant validity through two techniques named Fornell–Larcker criterion and heterotrait–monotrait (HTMT) (Hair Joe et al., 2016). In line with Fornell and Larcker (1981), the upper right-side diagonal values should be greater than the correlation with other variables, which is the square root of AVE, which indicates the discriminant validity of the model. Table 3 states that discriminant validity was developed top value of variable correlation with itself is highest. The HTMT ratios must be less than 0.85, although values in the range of 0.90 to 0.95 are appropriate (Hair Joe et al., 2016). Table 3 displays that all HTMT ratios are less than 0.90, which reinforces the statement that discriminant validity was supported in this study’s classification.

To determine the problem of multicollinearity in the model, VIF was calculated for this purpose. The experts said that if the value of VIF is greater than 5, there is no collinearity issue in findings (Hair et al., 2014). The results indicate that the inner value of VIF for all indicators must fall in the range of 1.421 to 1.893. Furthermore, these study findings show no issue of collinearity with data, and the study has stable results.

To evaluate “the explanatory power of the model,” the *R*^2^ value was analyzed for every predicted variable. It shows the degree to which independent variables illustrate the dependent variables. *R*^2^ value in “between 0 and 1 with higher values shows a higher level of predictive accuracy. Subsequent values of *R*^2^ describe 0.25 for weak, 0.50 for moderate, and 0.75 for” substantial. An appropriate model is indicated by *R*^2^ greater than 0.5 in primary results. In Figure 2, the value of *R*^2^ greater than 0.5 on all exogenous constructs, which also means that the model has strong predictive accuracy (Hair Joe et al., 2016).

Table 4 displays the percentage of variance clarified for every variable: 62.7% of continuous intention, 55.5% of participate intention, 54.5% for purchase intention, 80.9% for satisfaction, and 81.8% for social identification. In general, results demonstrate that values of *R*^2^ of endogenous variables are greater than 80%, which is the sign of a substantial “parsimonious model” (Sarstedt et al., 2014). Most importantly, the outputs give a significant validation of the model. *Q*^2^ values of all four 5 latent variables suggest that the model is extremely predictive (Hair et al., 2014).

**TABLE 4:** Predictive accuracy and relevance of the model.

Construct	R-square (R^2^)	(Q^2^)
Continuance intention	0.627	0.430
Participate intention	0.555	0.452
Purchase intention	0.545	0.395
Satisfaction	0.809	0.544
Social identification	0.818	0.512

### Hypothesis Testing

**FIGURE 3:**
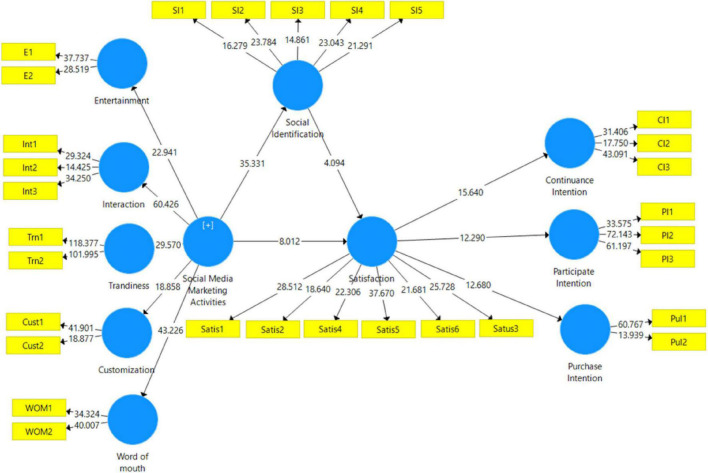
Structural model.

This study evaluates the significance of relationships using bootstrapping at 5,000 with a replacement sample (Hair Joe et al., 2016; Awan et al., 2021). The findings show that SMMAs have significant relationship with social identification (β = 0.905, *t*-value = 36.570, *p* = 0.000) which accept the H1. The findings show that SMM significantly influences the satisfaction (β = 0.634, *t*-value = 8.477, *p* = 0.000). Social identification has significant positive relationship with satisfaction as shown in Table 5 (β = 0.284, *t*-value = 4.348, *p* = 0.000) which accept the H3. The results show that satisfaction has significant relationship with continuous intention (β = 0.792, *t*-value = 15.513, *p* = 0.000) which support the H4. The findings show that satisfaction has strong positive relationship with participant intention (β = 0.745, *t*-value = 12.041, *p* = 0.000), which support the H5. The findings show that satisfaction has strong positive relationship with purchase intention (β = 0.739, *t*-value = 12.397, *p* = 0.000) which support the H6. The findings of the current investigation support H1, H2, H3, H4, H5, and H6. The results show that H4, H1a, H1b, H3a, H3b, H2a, and H2b are accepted (refer to Table 5 and Figure 3).

**TABLE 5:** Hypothesis testing.

	Hypothesis	Path coefficient (*t*-value)	Confidence interval	*F* square	*p*-values	Decision
H1	SMMA - > SI	0.905 (36.570)	0.838 to 0.937	4.507	0.000	Accepted
H2	SMMA - > Sat	0.634 (8.477)	0.443 to 0.789	0.383	0.000	Accepted
H3	SI- > Sat	0.284 (4.348)	0.137 to 392	0.077	0.000	Accepted
H4	Sat - > CI	0.792 (15.513)	0.791 to 0.872	1.678	0.000	Accepted
H5	Sat - > PI	0.745 (12.041)	0.596 to 0.835	1.246	0.000	Accepted
H6	Sat - > PUI	0.739 (12.397)	0.593 to 0.824	1.200	0.000	Accepted

Preacher and Hayes (2008) argue that if the VIF value is greater than 80%, then it shows full mediation, and value of VIF equal to 20 to 80% which indicate the partial mediation and if VIF falls below 20%, then there is no mediation. The findings show that social identification mediates the relationship between SMM and satisfaction (β = 0.213, *t*-value = 3.570, *p*-value = 0.000) and indirect effect (β = 0.257, *t*-value = 4.481, *p*-value = 0.000) with variance accounted for (VAF) 75% which show partial mediation. In this, the VAF describes the size of the indirect effect in relation to the total effect (Hayes and Preacher, 2010). The findings show that satisfaction mediates the relationship between social identification and continuous intention (β = 0.342, *t*-value = 3.435, *p*-value = 0.000) and indirect effect (β = 0.225, *t*-value = 4.636, *p*-value = 0.000) with VAF 64% which show partial mediation. In this, the VAF describes the size of the indirect effect in relation to the total effect (Hayes, 2009). The findings show that satisfaction mediates the relationship between social identification and participant intention (β = 0.324, *t*-value = 5.325, *p*-value = 0.000) and indirect effect (β = 0.211, *t*-value = 4.338, *p*-value = 0.000) with VAF 73% which show partial mediation. The findings show that satisfaction mediates the relationship between social identification and purchase intention (β = 0.312, *t*-value = 3.434, *p*-value = 0.000) and indirect effect (β = 0.3.213, *t*-value = 5.437, *p*-value = 0.000) with VAF 78% which show partial mediation (refer to Table 2).

## Discussion and Conclusion

The study was about SMMAs as proposed by Kim and Ko (2012), and it investigated which factors influence social media usage. The findings of the study include the following:

Most studies about social websites have not exhausted the impact of SMMAs. According to this study, SMMAs significantly affect social identification, which ultimately influences purchase decisions, participation decisions, continuance intention, and satisfaction. The study demystified social media usage intention. The findings were that SMMAs could sustain corporate brands. According to Beig and Khan (2018), unlike blog marketing and keyword advertising that were associated with content, SMM gets to the targeted audiences to enhance the impact of the information being shared by creating strong relationships in the online community. Therefore, service providers of social media must put into consideration means of increasing the impact of SMMAs. To boost SMMAs, operators should increase activity on the forum. The members of a community can be allowed to explain the guiding factors behind choosing a particular brand over that of competitors for other members to know the competing brands. From the discussions and sharing of knowledge, members get an opportunity to understand why they like a particular brand, thus enhancing brand loyalty and community cohesion (Yadav and Rahman, 2017).

The study also confirmed that most administrators are concerned with the influence of brand community management in creating business advantage. According to Tarsakoo and Charoensukmongkol (2020), marketing strategies and tools have undergone tremendous changes since the inception of social media. Consumers no longer must rely on traditional media to acquire information about a product before making their purchase since social media can effectively and easily avail such information. For that reason, social media service providers must come up with effective measures of controlling publication timing, frequency, and content to achieve the set marketing targets. According to this study, if a company can successfully assist users to easily identify with a particular brand community, strong relationships will be fostered between the consumers and the brand, hence creating customer’s loyalty (Ebrahim, 2020). Besides, users may stop using competitors’ products. So, companies need to appreciate that proper management of online strategies and brand community in creating community identity enhances brand’s competitiveness and inspires members of the brand community to shun using goods and services from competitors.

### Limitations and Recommendations

Regardless of the efforts geared toward enabling in-depth data collection, research methodology, and research structure, there were still various limitations that ought to be dealt with in studies to be conducted in the future. For instance, using online questionnaires in data collection, some members might have been very willing to fill them because of their community identity, hence enabling self-selection bias that may impact the validity and authenticity of the outcomes. Besides, a cross-sectional sample was used in the study; hence, results from the analysis can only demystify individual usage patterns on well-known social media. Nevertheless, the different social media platforms provide different services; hence, long-term usage needs long-term observation. The outcomes of growth model analysis with the experimental values and browsing experiences of users at the various phases in longitudinal studies to be conducted in the future may be increasingly conclusive on casual relationships with variables. The third limitation of the study is that different countries or areas have different preferences regarding social media. Future studies should unravel the reasons behind individuals from various cultural backgrounds or countries using different social media platforms and what might be the demands and motivations behind their preferences. Besides, new social networking sites such as Facebook and Twitter have unique characteristics which are different from traditional sites. Future studies should also focus on this shift. For this study, the emphasis was on SMMAs’ influence on user’s behavior and usage demands.

## Data Availability Statement

The raw data supporting the conclusion of this article will be made available by the authors, without undue reservation.

## Author Contributions

All authors listed have made a substantial, direct, and intellectual contribution to the work, and approved it for publication.

## Conflict of Interest

The authors declare that the research was conducted in the absence of any commercial or financial relationships that could be construed as a potential conflict of interest. The reviewer ZA declared a shared affiliation with one of the authors, SG, to the handling editor at time of review.

## Publisher’s Note

All claims expressed in this article are solely those of the authors and do not necessarily represent those of their affiliated organizations, or those of the publisher, the editors and the reviewers. Any product that may be evaluated in this article, or claim that may be made by its manufacturer, is not guaranteed or endorsed by the publisher.
